# Self-assembled silver nanoparticles monolayers on mica-AFM, SEM, and electrokinetic characteristics

**DOI:** 10.1007/s11051-013-1460-5

**Published:** 2013-02-09

**Authors:** Magdalena Oćwieja, Maria Morga, Zbigniew Adamczyk

**Affiliations:** Jerzy Haber Institute of Catalysis and Surface Chemistry, Polish Academy of Sciences, Niezapominajek 8, 30-239 Cracow, Poland

**Keywords:** Adsorption, Heterogeneous surfaces, Zeta potential, Streaming potential, Silver monolayers

## Abstract

A monodisperse silver particle suspension was produced by a chemical reduction method in an aqueous medium using sodium citrate. The average particle size determined by dynamic light scattering (DLS), transmission electron microscopy (TEM), and atomic force microscopy (AFM) was 28.5 nm. The DLS measurements confirmed that the suspension was stable for the ionic strength up to 3 × 10^−2^ M NaCl. The electrophoretic mobility measurements revealed that the electrokinetic charge of particles was negative for pH range 3–10, assuming −50 *e* for pH = 9 and 0.01 M NaCl. Using the suspension, silver particle monolayers on mica modified by poly(allylamine hydrochloride) were produced under diffusion-controlled transport. Monolayer coverage, quantitatively determined by AFM and SEM, was regulated within broad limits by adjusting the nanoparticle deposition time. This allowed one to uniquely express the zeta potential of silver monolayers, determined by the in situ streaming potential measurements, in terms of particle coverage. Such dependencies obtained for various ionic strengths and pH, were successfully interpreted in terms of the 3D electrokinetic model. A universal calibrating graph was produced in this way, enabling one to determine silver monolayer coverage from the measured value of the streaming potential. Our experimental data prove that it is feasible to produce uniform and stable silver particle monolayers of well-controlled coverage and defined electrokinetic properties.

## Introduction

On the last few years, silver nanoparticle have been extensively studied due to their potential applications in emerging areas of nanoscience and technology. They are widely applied in catalysis (Jiang et al. 2005), microelectronics (Li et al. 2005; Magdassis et al. 2003) or in medicine as antibacterial agents (Kim et al. 2007). Also, due to the efficient surface plasmon resonance, silver particles are used in surface-enhanced Raman spectroscopy (SERS) (Kaczor et al. 2010) and metal-enhanced fluorescence (MEF) (Aslan et al. 2005).

Silver nanoparticles are also frequently used to modify various organic and inorganic substrates to obtain materials of novel properties. However, most of practical applications of silver nanoparticles involve thin films deposited on various solid surfaces. Such films can serve as catalytic materials (Yougen et al. 2012), anti-reflecting layers (Kachan et al. 2006 coatings), antibacterial coatings (Lee et al. 2003), and active substrates for spectroscopy (Lee et al. 1997).

These thin films are usually produced via physical methods such as evaporation, sputtering (Arbab 2001), chemical vapor deposition (CVD) (Yuan et al. 1995), chemical bath deposition (CBD) (Estrada-Raygoza et al. 2006), lithographical techniques (Green and Liu 2003), electroless plating (Kim et al. 2008) and the sonoprocess method (Inoue et al. 2010). However, these techniques are expensive and require sophisticated equipment. Moreover, the purity and stability of silver films obtained in this way, are not satisfactory. Other disadvantages include a poor control of coverage and particle homogeneity within monolayers. To avoid such problems, the colloid self-assembly methods based on diffusion-controlled deposition of silver particles from stable suspensions can be used. This allows one to produced homogeneous, dense, almost defect-free, and ultrathin films (Mittler 2010). In this way, monolayers of controlled coverage and homogeneity can be produced by adjusting the suspension concentration, pH, ionic strength, and the deposition time (Brouwer et al. 2003; Kleimann et al. 2006; Oćwieja et al. 2011, 2012a, b). However, in these works indirect ex situ methods of particle monolayer characteristics such as UV–Vis surface plasmon adsorption (Bar et al. 1996), atomic force microscopy (AFM) (Yang et al. 2007), scanning electron microscopy (SEM), quartz crystal microgravimetry (QCM) (Bandyopadhyay et al. 1997), ellipsometry and reflectometry (Reiter et al. 1992) were used.

Therefore, the main goal of this work is to develop an efficient method for the in situ characterization of silver particle monolayers on solid substrates based on the streaming potential measurements. The method allows one to directly determine the kinetics of particle adsorption and desorption under various transport conditions such as diffusion and forced convection (flow). Thorough electrokinetic characteristics of the monolayers formed in this way can be exploited to determine their stability against pH cycling and ionic strength variations. This has major significance for optimizing the conditions of stable silver nanoparticle film preparation. It should be mentioned that up to our knowledge no such electrokinetic characteristics of silver particle monolayers on solid substrates have been reported in the literature.

## Experimental

### Materials

All chemical reagents used in the experiments (silver nitrate, trisodium citrate, sodium chloride, sodium hydroxide, and hydrochloric acid) were commercial products of Sigma Aldrich and used without further purification. The cationic polyelectrolyte, poly(allylamine hydrochloride) (PAH), having a molecular weight of 70 kDa was purchased from Polysciences and was used for mica surface modification. Natural ruby mica sheets obtained from Continental Trade was used as a substrate for the colloidal particle adsorption. The solid pieces of mica were freshly cleaved into thin fragments of desired area and used in each experiment without any pretreatment. Ultrapure water, used throughout this investigation, was obtained using the Milli-Q Elix&Simplicity 185 purification system from Millipore SA Molsheim, France.

### Synthesis of silver nanoparticles

Silver nanoparticles were obtained by a chemical reduction using trisodium citrate, which has been widely applied for the preparation of various metallic nanoparticles (Kamyshny and Magdassis 2009). An essential advantage of using sodium citrate is that it acts both as a reducing and stabilizing agent. In our work, the reaction was carried out at lower temperature and a shorter period of time than it is commonly applied. This allowed to produce monodisperse suspension of smaller particle than those described in the literature (Lee and Meisel 1981; Pillai and Kamat 2004).

A sample of silver nitrate (200 mg) was dissolved in distilled water to obtain the silver ions concentration of 1.18 mM and then heated rapidly to 88 °C under stirring (the rate of stirring 300 rpm). Afterward, 4 ml of 39 mM trisodium citrate solution was added rapidly to the silver solution. The mixture was kept at 88 °C for 35 min with continuous stirring. After this period of time, the silver sol was immediately cooled to the room temperature. Afterwards, the sol was purified from ionic excess using a stirred membrane filtration cell (Millipore, model 8400) with a regenerated cellulose membrane (Millipore, NMWL: 100 kDa). The washing procedure was repeated until the conductivity of the supernatant solution stabilized at ca. 15–20 μS cm^−1^.

### Preparation of silver nanoparticle monolayers

The procedure of preparing silver nanoparticle monolayers on PAH-modified mica was as fallows. First, a saturated PAH monolayer on mica was deposited under diffusion-controlled transport conditions. In order to do this, freshly cleaved mica sheets were placed in the PAH solution, having a concentration of 5 mg L^−1^, *I* = 10^−2^ M NaCl and pH = 4, and left for 15 min. Afterward, mica sheets covered by the polyelectrolyte monolayer were rinsed with water and immersed in the silver suspension.

Deposition of silver nanoparticles was carried out under diffusion-controlled transport from suspensions of different bulk concentrations (10, 25, 50, 100, 150 mg L^−1^), pH = 5.9, and various ionic strengths, varying from 10^−4^ to 3 × 10^−2^ M. The deposition time was regulated to obtain silver monolayers of a desired coverage. After deposition, the samples were rinsed with the ultrapure water and air dried. All experiments were performed in a diffusion cells which were equipped with a special glass element that allows for a vertical immersion of mica sheets in the colloidal suspensions. The deposition process was carried out under thermostated conditions, and the temperature was kept constant at 293 K.

### Methods of silver particle and monolayer characterization

The weight concentration of the particle suspension was determined by the high precision densitometer: DMA 5000M (Anton Paar). The UV–Vis extinction spectrum was measured using the Shimadzu UV-1800 spectrometer.

The silver particles were characterized by measuring their diffusion coefficient (*D*) and electrophoretic mobility (*μ*_e_) as a function of ionic strength (*I*) and pH. These parameters were acquired using the Zetasizer Nano ZS from Malvern (measurement range of 3 nm–10 μm for zeta potential, and 0.6 nm–6 μm for particle size).

The morphology of silver nanoparticles was investigated using the JEOL JSM-7500F microscope working in transmission mode. Samples for this examination were prepared by dispersing a drop of the silver colloid on a copper grid which was covered by a carbon film. Furthermore, the scanning electron microscope JEOL JSM-7500F was used to determine the coverage of silver monolayers. To insure a sufficient conductivity, the silver nanoparticles samples were covered with a thin layer of chromium.

Independently, the surface concentration of silver particles on the modified mica substrate was quantitatively determined using the atomic force microscopy (AFM). The measurements were carried out using the NT-MDT Solver Pro instrument with the SMENA SFC050L scanning head. Imaging was done in the semicontact mode using silicon probe (polysilicon cantilevers with resonance 120 kHz ± 10 %, typical tip curvature radius was 10 nm, contact angle <20°).

The zeta potential of bare, polyelectrolyte covered mica and silver monolayers of various coverage was determined via streaming potential measurements using a home-made cell previously described (Zembala and Adamczyk 2000; Zembala et al. 2001). The main part of the cell was a parallel plate channel of dimensions 2*b*_c_ × 2 *c*_c_ × *L* = 0.027 × 0.29 × 6.2 cm, formed by mica sheets separated by a perfluoroethylene spacer. The streaming potential *E*_s_ was measured using a pair of Ag/AgCl electrodes as a function of the hydrostatic pressure difference Δ*P*, which was driving the electrolyte flow through the channel. The cell electric conductivity *K*_e_ was determined by Pt electrodes. Knowing the slope of the *E*_s_ versus Δ*P* dependence, the apparent zeta potential of substrate surface (*ζ*_i_) can be calculated from the Smoluchowski relationship

1 $$ \zeta_{\text{i}} = \frac{\eta L}{{4\varepsilon b_{\text{c}} c_{\text{c}} R_{\text{e}} }}\left( {\frac{{\Updelta E_{\text{s}} }}{\Updelta P}} \right) = \frac{{\eta K_{\text{e}} }}{\varepsilon }\left( {\frac{{\Updelta E_{\text{s}} }}{\Updelta P}} \right) $$

where *η* is the dynamic viscosity of the solution, *ε* is the dielectric permittivity, *R*_e_ is the electric resistance of the cell governed mainly by the specific conductivity of the electrolyte in the cell.

## Results and discussion

### Bulk silver particle characteristics

The concentration of purified silver suspension was determined by a densitometer as described in our previous works (Oćwieja et al. 2011, 2012a, b). The sol was first purified using the membrane filtration method, then the specific density of the concentrated sol and its supernatant was measured. The silver sol having the concentration of 550 mg L^−1^ was then diluted (usually 10–150 mg L^−1^) and used for adsorption experiments.

**Fig. 1:**
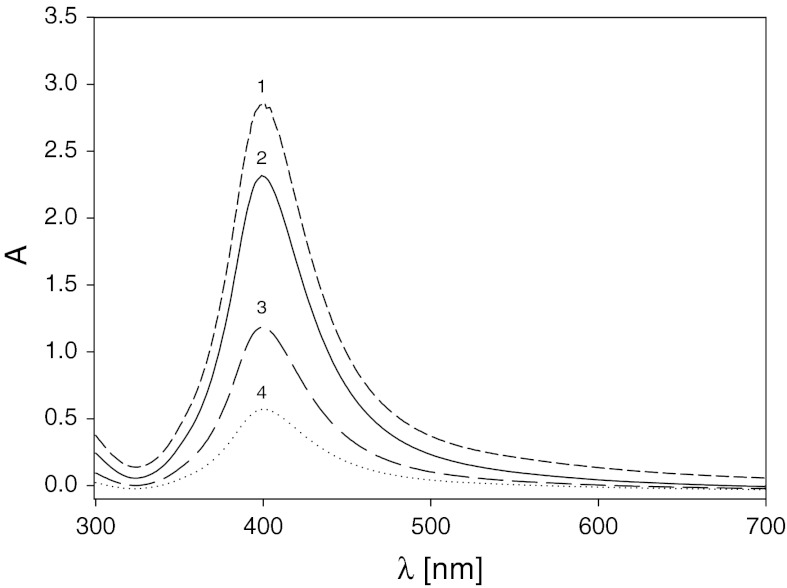
The UV–Vis absorption spectra of the aqueous silver nanoparticle suspension for various bulk concentrations *1* (*dash line*) 25 mg L^−1^, *2* (*straight line*) 20 mg L^−1^, *3* (*spaced dash line*) 10 mg L^−1^, *4* (*dotted line*) 5 mg L^−1^. The peak of the maximum spectrum extinction occurs at *λ*_max_ = 400 nm

The silver sol shows a single visible excitation band near 400 nm, attributed to the surface plasmon excitation of silver nanoparticles (Mie 1908), see Fig. 1. The plasmon band is narrow and symmetric, which indicates that the sample does not contain agglomerated particles (Henglein and Giersig 1999; Widoniak et al. 2005). The height of the peak increases monotonically with the sol concentration.

The size distribution and morphology of the silver particles were determined from AFM images and TEM micrographs. The nanoparticle diameter (*d*_p_) was calculated as the average value from two perpendicular directions and from the surface area of particles, as described before (Oćwieja et al. 2011, 2012a, b). According to the histogram obtained from TEM micrographs, the mean diameter of particles was 28 nm with a standard deviation of 4 nm.

The average size distribution of particles was also determined by AFM. Silver particles were deposited from a diluted suspension (20 mg L^−1^), pH = 5.5, *I* = 10^−2^ M for 15 min, on a mica sheet precovered by a supporting PAH layer. The size of the particles was determined using the Nova 1152 software which is coupled directly with AFM microscope. The average size of particles determined from the histogram was 29 nm, with a standard deviation of 5 nm.

The average particle size (hydrodynamic diameter, *d*_H_) was also determined via diffusion coefficient (*D*) measurements performed using the dynamic light scattering (DLS) method. Knowing the diffusion coefficient of particles, one can determine the hydrodynamic diameter using the Stokes–Einstein relationship

2 $$ d_{{H}} = \frac{kT}{3\pi \eta D} $$

where *k* is the Boltzman constant, *T* is the absolute temperature and *η* is the dynamic viscosity of the solution. The hydrodynamic diameter can be interpreted as the size of an equivalent sphere having the same hydrodynamic resistance coefficient as the particle. The advantage of using this quantity in comparison to the diffusion coefficient, is that it is independent on temperature and liquid viscosity, so it can be used for analyzing the suspension stability under various conditions. For sake of convenience, silver particle size, determined using different techniques are collected in Table 1.

**Table 1:** Physicochemical characteristics of silver nanoparticles

Property	Value	Remarks
Specific density (g cm^−3^)	10.49	Literature data (Fuertes et al. 2009)
Average particle size (nm)	28 ± 4	From size distribution derived from TEM micrographs
Average particle size (nm)	29 ± 5	From size distribution derived from AFM images
Diffusion coefficient (cm^2^ s^−1^)	1.48 × 10^−7^	Determined by DLS for *T* = 293 K, pH = 6.2 *I* = 5 × 10^−2^–0.03 M NaCl
Hydrodynamic diameter (nm)	29 ± 5	Calculated from Eq. (2)
Average particle size (nm)	28.4	Calculated from adsorption kinetics Eq. (11)
Geometrical cross-section area (nm^2^)	615	Calculated from geometry
Plasmon absorption maximum (nm)	400	Measured for pH = 6.2 *I* = 10^−2^ M NaCl and silver sol concentration *c*_b_ = 5–25 mg L^−1^

**Fig. 2:**
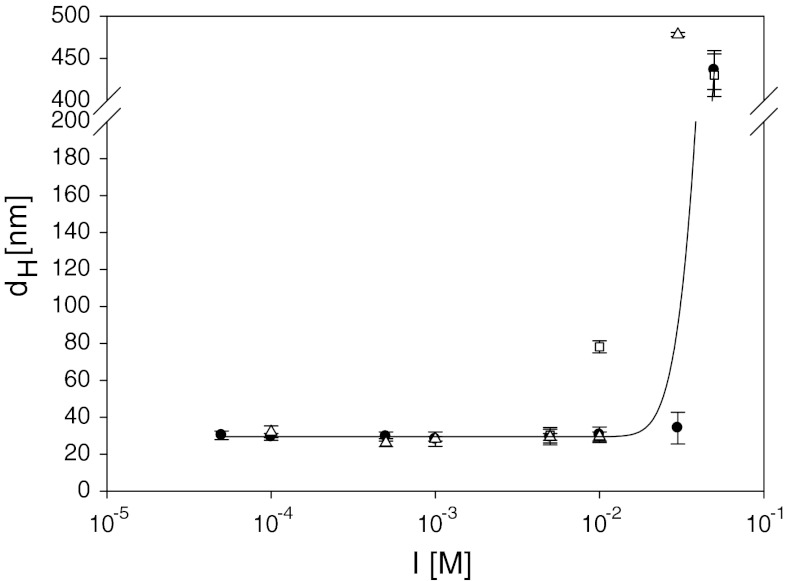
The dependence of the hydrodynamic diameter of silver nanoparticles on ionic strength, determined by DLS (*open square*) pH = 3, (*filled circle*) pH = 6.2, (*open triangle*) pH = 9, sol concentration 100 mg L^−1^

The stability of the silver sol was studied by determining the dependence of the hydrodynamic diameter of particles on ionic strengths varied between 5 × 10^−5^ and 10^−1^ M NaCl for various pH (see Fig. 2).

As can be seen, for ionic strengths up to 3 × 10^−2^ M and pH = 6.2, the hydrodynamic diameter was practically constant, attaining an average value of 29 nm with a standard deviation of 5 nm. The value is in accordance with that previously determined by AFM. A significant increase in the hydrodynamic diameter was observed for ionic strengths higher than 0.05 M.

**Fig. 3:**
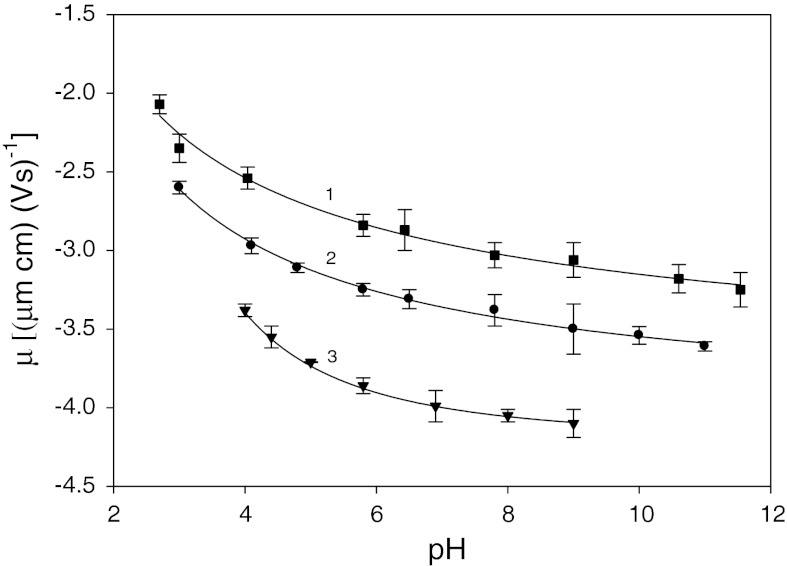
The dependence of the electrophoretic mobility of silver particles on pH *1* (*filled* *square*) *I* = 10^−2^ M, *2* (*filled circle*) *I* = 10^−3^ M, *3* (*filled down-pointing triangle*) *I* = 10^−4^ M. The *solid lines* are a guide to the eye

Another important parameter, which describes the electrokinetic charge of particles and the electrostatic interaction among them, is the electrophoretic mobility *μ*_e_, defined as the average translation velocity of colloidal particles under given electric field. The dependence of the electrophoretic mobility of silver nanoparticles on pH was shown in Fig 3.

The electrophoretic mobility of silver nanoparticles is negative in entire range of ionic strength and pH, indicating that the particles acquired a net negative charge. It increases with ionic strength, from −3.38 μm cm (V s)^−1^ for *I* = 10^−4^ M to −2.51 μm cm (V s)^−1^ for *I* = 10^−2^ M and pH = 4. Higher negative charges were observed for lower ionic strengths and higher pH values. For *I* = 10^−3^ M and pH = 9, *μ*_e_ = −3.48 μm cm (V s)^−1^, and for *I* = 10^−4^ M and pH = 9, *μ*_e_ = −4.08 μm cm (V s)^−1^.

Knowing the electrophoretic mobility of particles, one can calculate the average number of charges per particle from the Lorentz–Stokes relationship (Dąbkowska and Adamczyk 2012; Wasilewska and Adamczyk 2011)

3 $$ N_{\text{c}} = \frac{30\pi \eta }{1.602}d_{\text{H}} \mu_{\text{e}} $$

where *N*_*c*_ is expressed as the number of elementary charge (*e*) per particle (*e* = 1.602 · 10^−19^ C), η is expressed in g (cms)^−1^ *d*_H_ is the particle diameter expressed in *nm* and *μ*_*e*_ is expressed in *μ*m cm (V s)^−1^.

For pH 6.2 and the ionic strength equal to 10^−3^, 10^−2^, and 3 × 10^−2^ M, the number of uncompensated (electrokinetic) charges was −54*e*, −47*e*, and −44*e*, respectively.

Knowing the electrophoretic mobility, this quantity can be calculated using Henry’s equation

4 $$ \zeta_{\text{p}} = \frac{\eta }{{\varepsilon f(\kappa d_{\text{p}} )}}\mu_{\text{e}} , $$

where *ζ*_p_ is the zeta potential of particles, *ε* is the electric permittivity of the solution, *f*(*κd*_p_) is the correction function of the dimensionless parameter, *κd*_p_, $\kappa^{ - 1} = \left( {\frac{\varepsilon kT}{{2e^{2} I}}} \right)^{1/2}$ is the thickness of the electric double layer. For thin double layers (*κd*_p_ > 10), *f*(*κd*_p_) approaches unity (Smoluchowski’s approximation), and for thick double layers (*κd*_p_ > 1), *f*(*κd*_p_) approaches ^3^/_2_ (Hückl approximation). Moreover, in our calculations of the zeta potential, the Ohshima’s model was used (Ohshima 2006) which includes the relaxation effect of double layer around particles especially for low ionic strengths when the parameter *κd*_p_ attains lower values. The zeta potentials of the particles, calculated from three models, are given in the Table 2.

**Table 2:** The electrophoretic mobility, the number of elementary charge and the zeta potentials of silver nanoparticles for various pH and ionic strength

pH	Ionic strength (M)	*κd*_p_	*μ*_e_, μm cm (V s)^−1^	*N*_c_ (*e*)	*ζ*_p_ (mV) Smoluchowski’s model	*ζ*_p_ (mV) Henry model	*ζ*_p_ (mV) Ohshima’s model
3	0.001	1.45	−2.60	−43	−36.6	−52.4	−59.2
	0.01	4.59	−2.35	−40	−33.1	−43.6	−48.1
	0.03	7.95	−2.19	−36	−30.8	−37.9	−40.3
6.2	0.0001	0.46	−3.86	−64	−54.4	−80.5	−95.1
	0.001	1.45	−3.25	−54	−45.8	−65.5	−87.3
	0.01	4.59	−2.84	−47	−40.0	−52.7	−62.8
	0.03	7.95	−2.72	−44	−38.3	−47.1	−52.3
9	0.0001	0.46	−4.10	−68	−57.7	−85.5	−105
	0.001	1.45	−3.50	−58	−49.3	−70.5	–*
	0.01	4.59	−3.06	−50	−43.1	−56.8	–*

*Oshima model fails

From these measurements one can conclude that the silver particles exhibit a high negative zeta potential for a broad range of pH and ionic strength, which is expected to promote their efficient deposition on positively charged substrates.

### Substrate characteristics

**Fig. 4:**
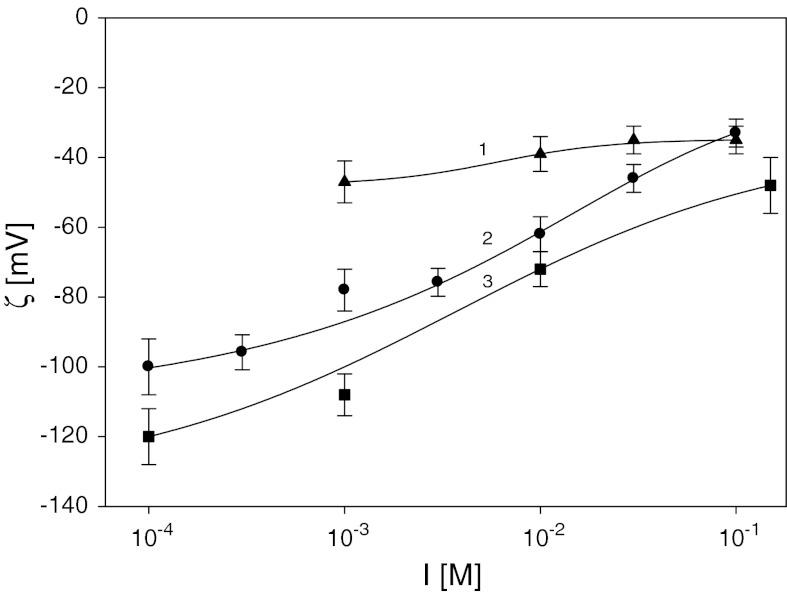
The dependence of the apparent zeta potential of mica on ionic strength *1* (*filled triangle*) pH = 3.5, *2* (*filled circle*) pH = 5.5, *3* (*filled square*) pH = 9.0 determined by the streaming potential method. The *solids lines* are a guide to the eye

Except for particle characteristics in the bulk, a proper interpretation of the adsorption phenomena requires a quantitative information about the substrate zeta potential. As mentioned, in our experiments, mica sheets modified by PAH were used as a well-defined substrate. Mica is a chemically stable and molecularly smooth material, characterized by a uniform and homogeneous surface charge distribution. The zeta potential of a bare mica substrate (*ζ*_i_) for various ionic strengths and pH, determined using the streaming potential method is shown in Fig. 4. As seen, the zeta potential *ζ*_i_ attained high negative values in the entire range of pH and ionic strength reaching −120 mV for pH = 9 and *I* = 10^−4^ M, −62 mV for pH = 5.5 and *I* = 10^−2^ M, and −37 mV for pH = 3.5 and *I* = 3 × 10^−2^ M.

**Fig. 5:**
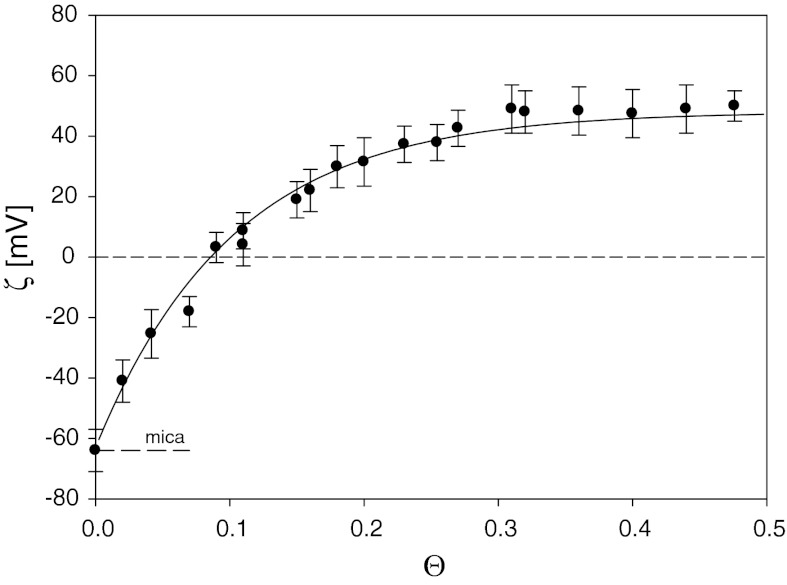
The dependence of the zeta potential of mica *ζ* on the coverage of PAH *Θ*, (*filled circle*) (*I* = 10^−2^ M, pH = 5.5) determined by the streaming potential method. The *solid line* denotes the theoretical results calculated from the electrokinetic model, Eqs. (5), (6)

To promote an efficient particle deposition, the negative zeta potential of mica was converted to positive by controlled adsorption of PAH molecules, which is a popular and useful method of surface functionalization (Lowack and Helm 1995; Oćwieja et al. 2011; Schmitt et al. 1999). The quantitative analysis of PAH monolayer formation on the mica substrate was investigated using the streaming potential method, according to the procedure described in our previous works (Adamczyk et al. 2006; Oćwieja et al. 2011). The amount of the adsorbed polyelectrolyte was regulated by changing its bulk concentration and adsorption times. The dependence of the apparent zeta potential on PAH coverage at ionic strength *I* = 10^−2^ M is shown in Fig. 5.

The coverage of PAH monolayers was regulated by changing the bulk concentration of polyelectrolyte solutions (1–10 mg L^−1^) and the adsorption time (5–30 min). As seen, the zeta potential of mica increases abruptly with the coverage of PAH. The most significant change is observed for low coverage up to 0.1, when the slope of *ζ* versus *Θ* dependence exceeds 10. For higher coverage of PAH, *Θ* > 0.25, the zeta potential variations become rather minor. Thus, for *Θ* > 0.3 limiting zeta potentials of PAH are attained. As proved in our previous works (Adamczyk et al. 2006), for dense monolayers formed, the asymptotic values of the zeta potential approach 1/√2 = 0.71 of the bulk zeta potential of the particles or polyelectrolyte molecules. The experimental results shown in Fig. 5, were interpreted in terms of the electrokinetic model in which adsorbed molecules are treated as isolated entities exhibiting a 3D charge distribution (Adamczyk et al. 2010; Adamczyk et al. 2011; Morga et al. 2012).

Using this approach, the expression for the streaming potential of an interface covered by particles can be formulated in the analytical form

5 $$ \zeta (\Uptheta ) = F_{\text{i}} (\Uptheta )\zeta_{\text{i}} + F_{\text{p}} (\Uptheta )\zeta_{\text{p}} $$

where *ζ*(*Θ*) is the zeta potential of the particle covered substrate and $F_{\text{i}} \left( \Uptheta \right),\,F_{\text{p}} \left( \Uptheta \right)$ are the dimensionless functions of the particle coverage and the *κd*_*p*_ parameter.

The $F_{\text{i}} \left( \Uptheta \right)$ function reflects the effect of the flow damping in the vicinity of deposited particles, which diminishes the rate of ion transport (convective current) and the $F_{\text{p}} \left( \Uptheta \right)$ function describes the convective current from the electric double layer at particles (Adamczyk et al. 2010).

It should be mentioned that Eq. (5) does not involve any fitting parameters because the functions $F_{\text{i}} \left( \Uptheta \right),\,F_{\text{p}} \left( \Uptheta \right)$ were theoretically determined.

Theoretical results were also reported in the previous work (Adamczyk et al. 2010), which allowed one to determine the $F_{\text{i}} \left( \Uptheta \right),\,F_{\text{p}} \left( \Uptheta \right)$ functions for the entire range of coverage in the limit of thin double layers. These results were obtained by numerically evaluating the flow field in the vicinity of adsorbed particles using the multipole expansion method. The exact numerical results can be approximated by the following analytical interpolation functions, with a margin of error (±) 1 %

$$ F_{\text{i}} (\Uptheta ) = {\text{e}}^{{ - C_{\text{i}}^{0} \theta }} $$

6 $$ F_{\text{p}} (\Uptheta ) = \frac{1}{\sqrt 2 }(1 - {\text{e}}^{{ - \sqrt 2 C_{\text{p}}^{0} \theta }} ) $$

where the *C*_i_, *C*_p_ are the functions of *κa* parameter, attaining constant values of $C_{\text{i}}^{0} = 10.2$ and $C_{\text{p}}^{0} = 6.51$, for *κa* > 1.

Obviously, for bare surfaces, where *Θ* = 0, $F_{\text{i}} \left( \Uptheta \right)$ = 1 and $F_{\text{p}} \left( \Uptheta \right)$ = 0. On the other hand, for high coverage range the $F_{\text{i}} \left( \Uptheta \right)$ function vanishes and $F_{\text{p}} \left( \Uptheta \right)$ tends to $\frac{1}{\sqrt 2 }$. Thus, using Eq. (5) one can deduce that the limiting zeta potential for surfaces covered by particles is given by

7 $$ \zeta_{\infty } = \zeta_{\text{p}} /\sqrt 2 = \, 0. 70 1\zeta_{\text{p}} $$

Theoretical results calculated using Eqs. (5), (6) are plotted in Fig. 5 as a solid line. As can be seen, they properly reflect the experimental data for the entire coverage range. In particular, the validity of Eq. (7) for describing PAH monolayer behavior was confirmed.

**Fig. 6:**
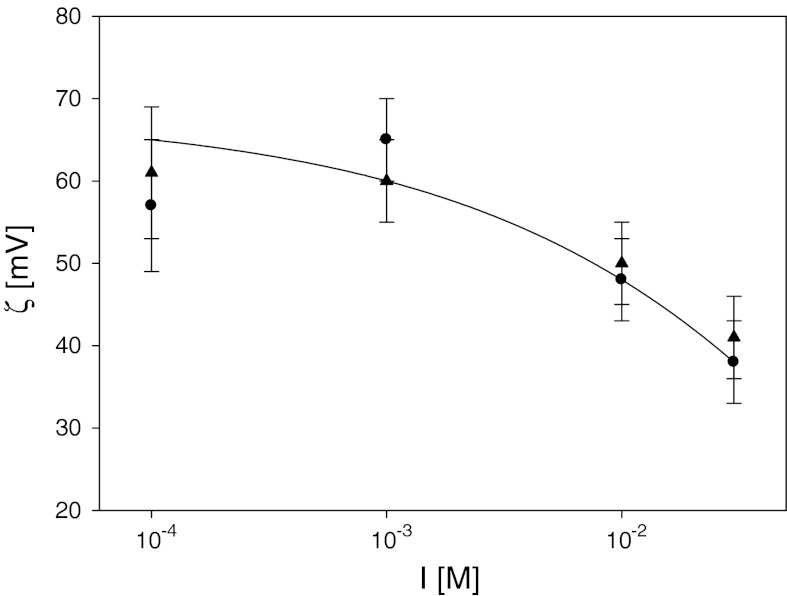
The dependence of the zeta potential of saturated PAH monolayers on ionic strength determined by the streaming potential method (*filled triangle*) pH = 3.5 (*filled circle*) pH = 5.5. The *solid line* is a guide to the eye

The dependence of this limiting value of the zeta potential of PAH monolayer is plotted in Fig. 6. As can be seen, it attains the value of 49 mV for *I* = 10^−2^ M and 60 mV for *I* = 10^−4^ M. Such high positive values of the zeta potential suggest that an efficient deposition of negatively charged particles should take place. This was confirmed by particle deposition experiments discussed in the next section.

### Kinetics of silver particle deposition

Deposition of silver particles on PAH-modified mica was conducted under diffusion-controlled transport according to the procedure described above. The first step in these kinetic measurements was the deposition of a saturated PAH monolayer on mica sheets. Afterward, the particle deposition was carried out using silver sols of the concentration ranging from 5 to 150 mg L^−1^, for a desired time period ranging from 5 to 1500 min. The surface concentration of the deposited particles *N*_s_ was determined by direct enumeration, using AFM images and SEM micrographs. Usually, 8–10 various areas were considered with the overall number of particles counted about 1,000, which ensured the precision of these measurements better than 3 %. The results were expressed as the surface concentration of particles *N*_s_, defined as the number of particles per unit area of the substrate. For the sake of convenience, this area was assumed to be one square μm; thus *N*_s_ has the dimension of μm^−2^. Knowing the average number of silver particles per unit area, the size of the particle and the surface area Δ*S*, the surface concentration can be converted to dimensionless coverage defined as:

8 $$ \Uptheta = \frac{{{\pi}{d_{\text{p}}}^{2}N_{\text{s}} }}{4} $$

Knowing *N*_s_ as a function of deposition time, particle adsorption kinetics for various experimental conditions can be determined. However, it should be mentioned that for the diffusion-controlled transport, it is appropriate to express the kinetic runs in terms of the square root of deposition time *t* ^1/2^, rather than the primary time variable *t*. For a low coverage range, where the surface blocking effects remain negligible, the kinetics of particle adsorption is described by the theoretical formula (Adamczyk 2006)

9 $$  {N_{{S}} } = 2\left( {\frac{D}{\pi }} \right)^{1/2} t^{1/2} n_{{b}} $$

where *D* is the diffusion coefficient and *n*_b_ is the bulk number concentration of particles connected with the weight concentration *c*_p_ (expressed in mg L^−1^) via the linear dependence

10 $$ n_{{b}} = \frac{{6 \times 10^{ - 6} }}{{\pi {d_{{p}}}^{3}\rho_{{p}}  }}c_{{p}} $$

where $\rho_{\text{p}}$ is the specific density of silver (see Table 1).

**Fig. 7:**
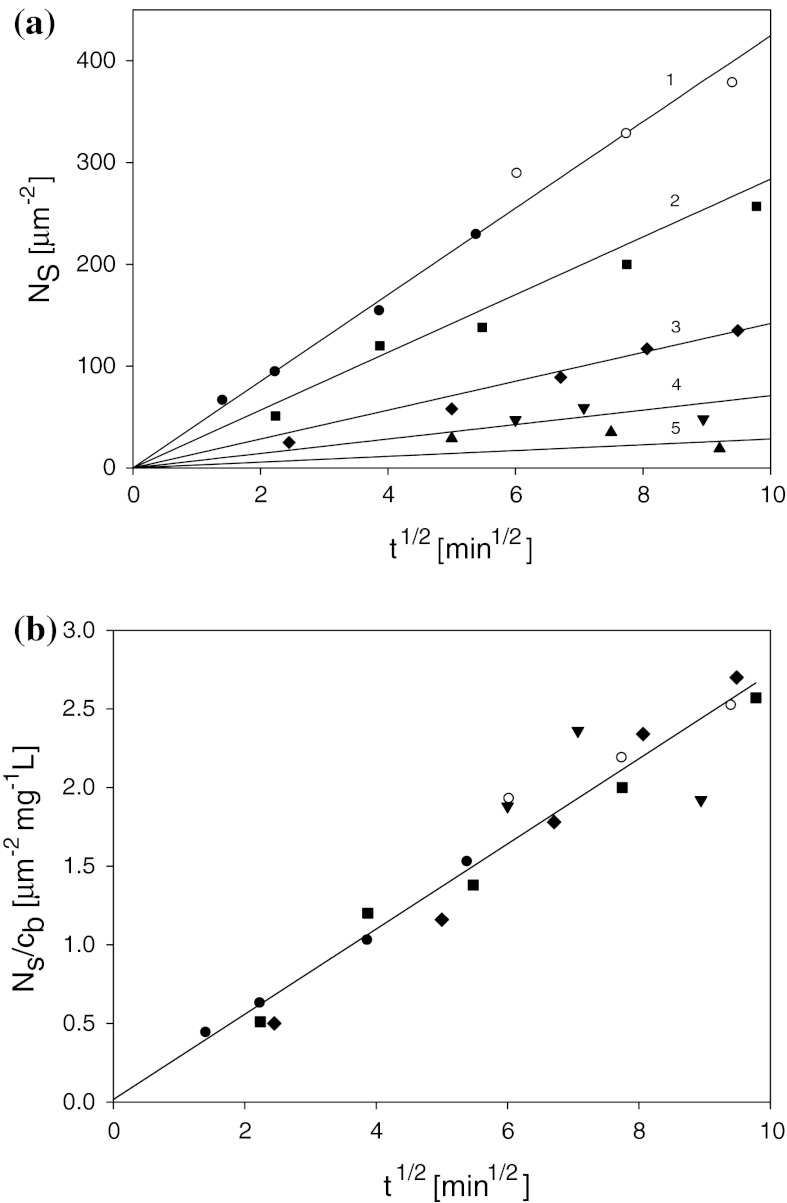
**a** The kinetics of silver particle adsorption on mica determined for various bulk suspension concentrations *1* (*filled circle*, *open* *circle*) 150 mg L^−1^, *2* (*filled square*) 100 mg L^−1^, 3 (*filled diamond*) 50 mg L^−1^, *4* (*filled down-pointing triangle*) 25 mg L^−1^, and *5* (*filled triangle*) 10 mg L^−1^. **b** The dependence of the reduced surface concentration *N*_s_/*c*_b_ (μm^−2^ mg L^−1^) on the square root of adsorption time *t* ^1/2^ (min^1/2^)

Typical kinetic runs obtained for a wide range of bulk silver particle concentration, 10–150 mg L^−1^ and deposition conditions *I* = 10^−2^ M NaCl, pH = 5.5, and *T* = 293 K are shown in Fig. 7a. As can be seen, for *t* ^1/2^ < 10 min^1/2^ (adsorption time *t* < 100 min) particle deposition was linear in this coordinate system, with the slope increasing proportionally to the bulk concentration.

This indicates that the dependence of *N*_s_/*c*_*b*_ on *t* ^1/2^ should yield a universal straight-line dependence as can be observed in Fig. 7b. The linear dependence of *N*_s_/*c*_b_ (mg L^−1^ μm^−2^) on *t* ^1/2^ (min^1/2^) exhibits the slope $s_{\text{D}} = {{\Updelta \left( {\frac{{N_{\text{s}} }}{{c_{\text{b}} }}} \right)} \mathord{\left/ {\vphantom {{\Updelta \left( {\frac{{N_{\text{s}} }}{{c_{\text{b}} }}} \right)} {\Updelta t^{1/2} }}} \right. \kern-0pt} {\Updelta t^{1/2} }}$ of 0.271 mg L^−1^ μm^−2^ min^−1/2^ = 3.49 × 10^12^ cm g^−1^ s^−1/2^. This confirms the proportionality of the particle adsorption rate to the bulk suspension concentration, in accordance with Eq. (9). Knowing the slope, one can experimentally determine the diameter of silver particles from the dependence previously derived (Oćwieja et al. 2012a, b):

11 $$ d_{\text{m}} = \left( {\frac{12}{{\rho_{\text{p}} \pi^{2} s_{\text{D}} }}} \right)^{2/7} \left( {\frac{kT}{3\eta }} \right)^{1/7} $$

In our case, using the above value of *s*_D_ and noting that *T* = 293 K, *η* = 0.01 g (cm s)^−1^ and *ρ*_p_ = 10.49 g cm^−3^, one obtains *d*_m_ = 28.4 nm from Eq. (11). As can be seen, the value of the “diffusion” diameter of particles determined from the adsorption kinetic experiments agrees with the values determined using dynamic light scattering or spectroscopic measurements (TEM, AFM), see Table 1.

**Fig. 8:**
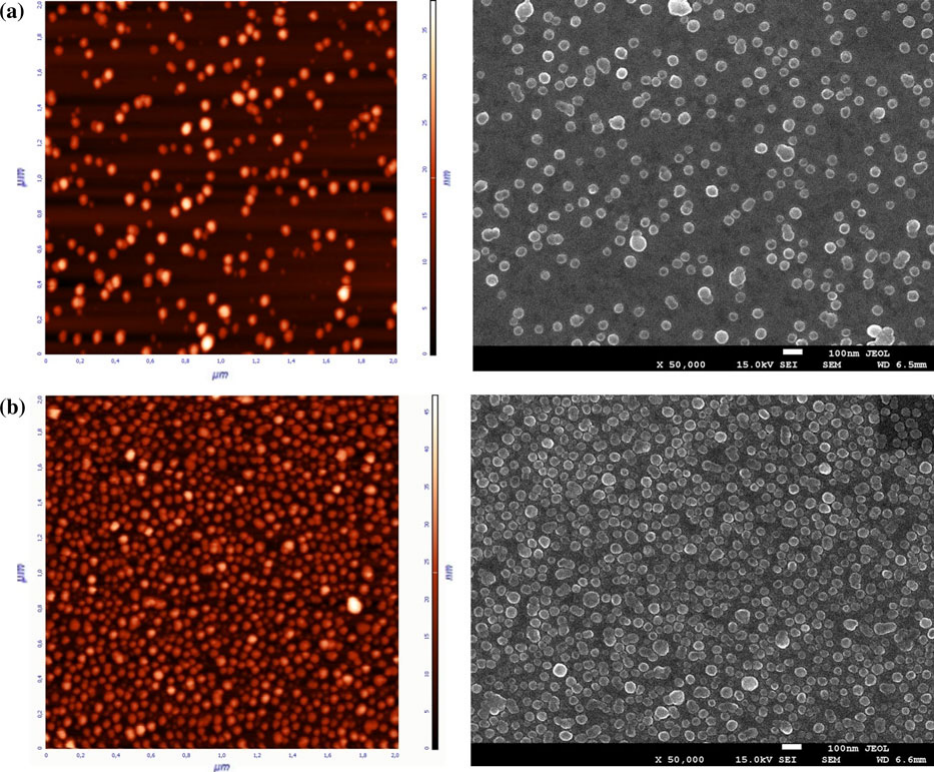
AFM images and SEM micrographs of silver particle monolayers adsorbed on mica modified by the saturated PAH monolayer. Particle deposition conditions *c*_p_ = 150 mg L^−1^, pH = 5.5, *I* = 10^−2^ M, **a** deposition time *t* = 2 min, *N*_s_ = 54 μm^−2^, *Θ* = 0.03, **b** deposition time *t* = 40 min, *N*_s_ = 276 μm^−2^, *Θ* = 0.17

In the next series of experiments, the kinetics of particle deposition for various ionic strengths was investigated for longer times with the aim of determining the maximum coverage of self-assembled monolayers. Particle monolayers formed under ionic strength 10^−2^ M, and pH = 5.5 for increasing deposition times are shown in Fig. 8.

**Fig. 9:**
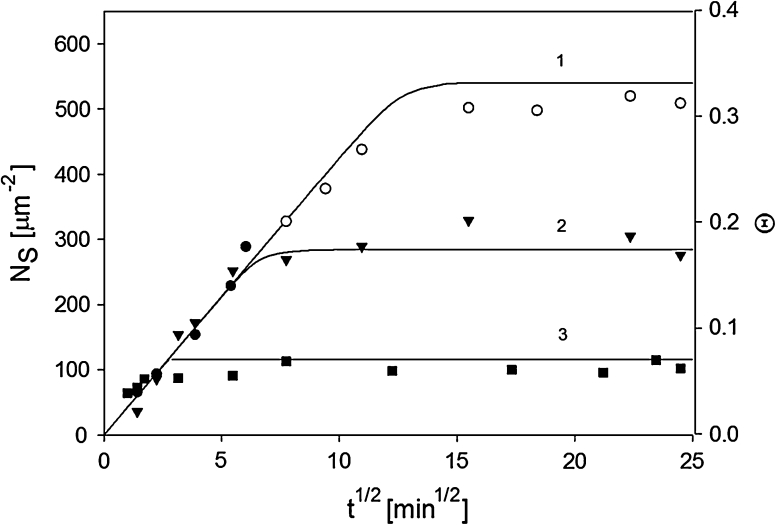
The kinetics of silver particle adsorption on mica determined for various ionic strengths *1* (*filled circle*, *open* *circle*) 10^−2^ M, *2* (*filled down-pointing triangle*) 10^−3^ M, *3* (*filled square*) 10^−4^ M determined using AFM (*full points*) and SEM (*hollow points*). Particle deposition conditions pH = 5.5, *T* = 293 K, *c*_b_ = 150 mg L^−1^. The *solid lines* denote the theoretical results calculated from the RSA model

In Fig. 9, kinetic runs obtained for various ionic strengths are shown. The surface concentration of particles was determined by both AFM (full points) and SEM (hollow points). Characteristic features of these kinetic runs are a linear increase in *N*_*s*_ with *t* ^1/2^ for shorter times and then, after reaching some critical time, an abrupt stabilization of the surface concentration at a constant value. This behavior was quantitatively described in terms of the random sequential adsorption (RSA) model depicted by the solid lines in Fig. 9. This model was applied before for describing irreversible adsorption (deposition) of colloid micro-particles (polystyrene latexes) (Adamczyk and Szyk 2000; Kleimann et al. 2006), nanoparticles (Oćwieja et al. 2012a, b; Pericet-Camora et al. 2004; Popa et al. 2007) and proteins (Dąbkowska and Adamczyk 2012). The RSA model is also extensively discussed in the book (Adamczyk 2006).

In Fig. 9, the maximum particle coverage *Θ*_mx_ systematically increases with ionic strength from 0.07 for *I* = 10^−4^ M to 0.33 for *I* = 10^−2^ M. It is worth mentioning, that for higher ionic strengths the silver suspension became unstable and the aggregation process was observed (see Fig. 2), therefore the adsorption cannot be studied for *I* > 10^−2^ M.

### Streaming potential of silver monolayer on PAH-modified mica

**Fig. 10:**
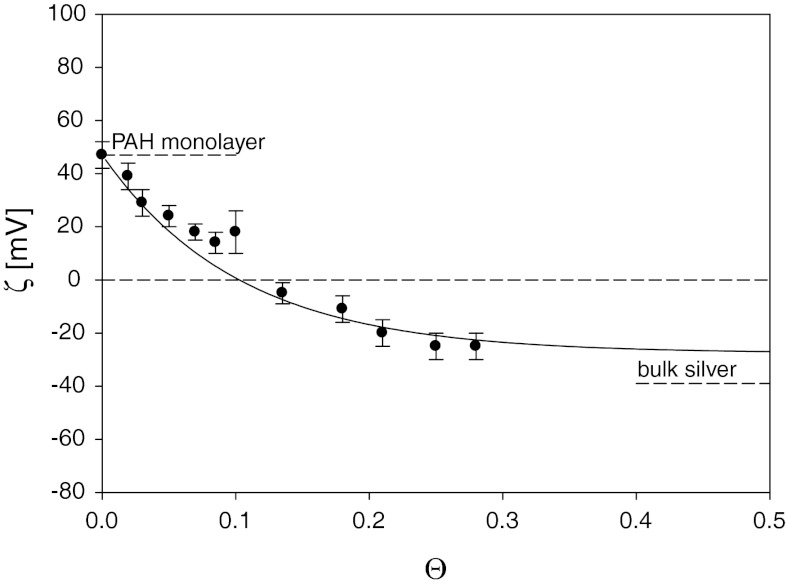
The dependence of the zeta potential of PAH monolayer *ζ* on the coverage of silver particles *Θ* determined by the streaming potential method. The deposition conditions *I* = 10^−2^ M NaCl, pH = 5.8

The disadvantage of the AFM and SEM methods is that they are working under ex situ conditions, which require drying of particle monolayers. This is eliminated in the streaming potential method, allowing for in situ measurements. The measurements were carried out according to the following procedure: in the first step, the streaming potential cell, assembled from two freshly cleaved mica sheets forming the channel, was filled in with the PAH solution (*c*_PAH_ = 5 mg L^−1^, *I* = 10^−2^ M NaCl). The PAH molecules were allowed to adsorb for a required time under diffusion-controlled transport forming a saturated monolayer. After the deposition was completed the cell was flushed with a pure electrolyte solution to remove weakly bonded particles and the streaming potential measurement was conducted in situ. The zeta potential of the mica covered by particles was calculated using the equation (Eq. 1). Knowing the zeta potential, one can control the coverage of PAH (see Fig. 5). Afterward, working still under in situ conditions, the procedure was repeated for the silver particles. To obtain a desired coverage, the amount of adsorbed particles was regulated by changing its bulk concentration (50–200 mg L^−1^) and keeping the desired adsorption time (1–40 min). The zeta potential of PAH-covered mica on the coverage of silver particles obtained for *I* = 10^−2^ M is shown in Fig. 10.

As seen, the formation of particle monolayer results in an abrupt decrease in the surface zeta potential with the slope *ζ* versus *Θ* considerably exceeding 10 for *Θ* close to 0.1. The inversion in sign of polyelectrolyte monolayer is observed for *Θ*_p_ = 0.1. For higher coverage of silver particles (*Θ* > 0.2), the zeta potential variations become rather minor and for

*Θ*_p_ > 0.25 the zeta potential of the silver layer attains the asymptotic value of −25 mV. Thus, it was confirmed that the limiting zeta potential *ζ* obtained for maximum coverage of silver nanoparticles is systematically lower than its bulk value equal to −39 mV. As proved in our previous works (Adamczyk et al. 2006; Zaucha et al. 2011), for dense particle monolayers, the asymptotic values of the zeta potential approaches 1/√2 = 0.701 of the bulk zeta potential of the nanoparticles. This was interpreted theoretically in terms of the electrokinetic model described above (Eq. 6). As seen in Fig. 10, the theoretical results obtained from Eqs. (5), (6), properly reflect the experimental data for the entire coverage range. To the best of our knowledge, this is the first result of this kind reported for conducting particles.

Furthermore, Eqs. (5) and (6) can be transformed to a useful, universal form by introducing the normalized zeta potential (Dąbkowska and Adamczyk 2012)

12 $$ \overline{\zeta } = \frac{{\zeta - \zeta_{\text{p}} /\sqrt 2 }}{{\zeta_{\text{i}} - \zeta_{\text{p}} /\sqrt 2 }} = \frac{{\zeta - \zeta_{\infty } }}{{\zeta_{\text{i}} - \zeta_{\infty } }} $$

Using this transformation, one can express the results obtained for various conditions (various ionic strength) through the relationship

13 $$ \bar{\zeta } = {e_i}^{{-C_i}{\Uptheta_{S}}} $$

It should be mentioned that Eq. (13) is universally valid for an arbitrary particle size, zeta potential of particles and substrates, pH, etc. In practice, the unknown coverage of the silver monolayer on an arbitrary substrate can be determined via the streaming potential measurements from the following relationship obtained by inverting Eq. (13)

14 $$ \Uptheta_{\text{S}} = - \frac{1}{{C_{\text{i}} }}\ln \frac{{\zeta - \zeta_{\infty } }}{{\zeta_{\text{i}} - \zeta_{\infty } }} $$

**Fig. 11:**
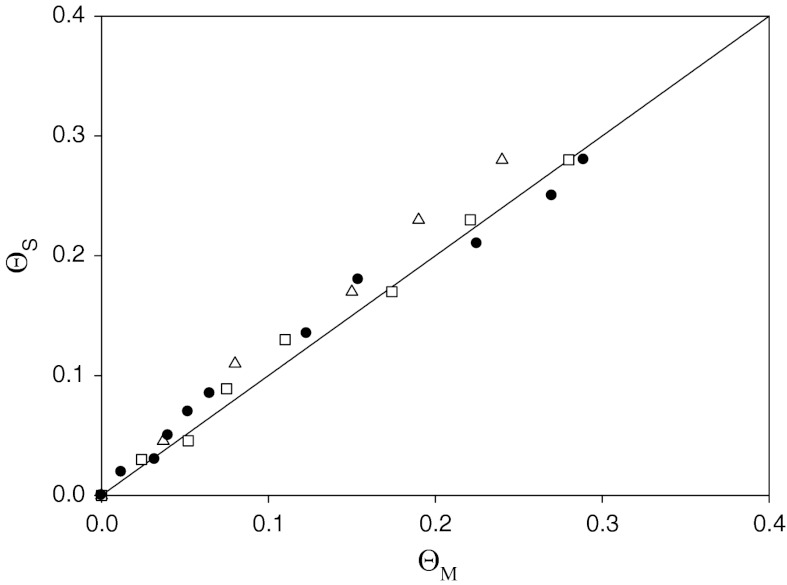
The universal plot dependence of the coverage of silver particles, calculated on the basis of the streaming potential measurements *Θ*_S_ from Eq. (13), on the coverage *Θ*_M_, obtained from the microscope images. The *points* denote experimental results obtained for various ionic strengths of the NaCl electrolyte *I* (*open triangle*) *I* = 10^−4^ M, (*open square*) *I* = 10^−3^ M, (*filled circle*) *I* = 10^−2^ M

Obviously, in order to apply Eq. (14), one should also know the zeta potential of the uncovered substrate *ζ*_i_ and the zeta potential of particles in the bulk *ζ*_p_. The results calculated this way are compared in Fig. 11 with results obtained from direct AFM and SEM determination of particle coverage *Θ*_M_.

As seen in Fig. 11, the results obtained from streaming potential studies, *Θ*_s_, are in a good agreement with those obtained from the microscope images *Θ*_M_. This is reflected by the slope of the regression being exactly one. This is of importance because these result prove that the streaming potential method is a useful tool not only for determining the zeta potential of particles but also for precisely calculating the coverage directly under wet, in situ conditions.

In addition, the streaming potential measurements create a unique possibility of thorough characterization of nanoparticle monolayers, for example, to determine the isoelectric point (Morga et al. 2012) or monolayer stability under various physicochemical conditions within a prolonged time period.

**Fig. 12:**
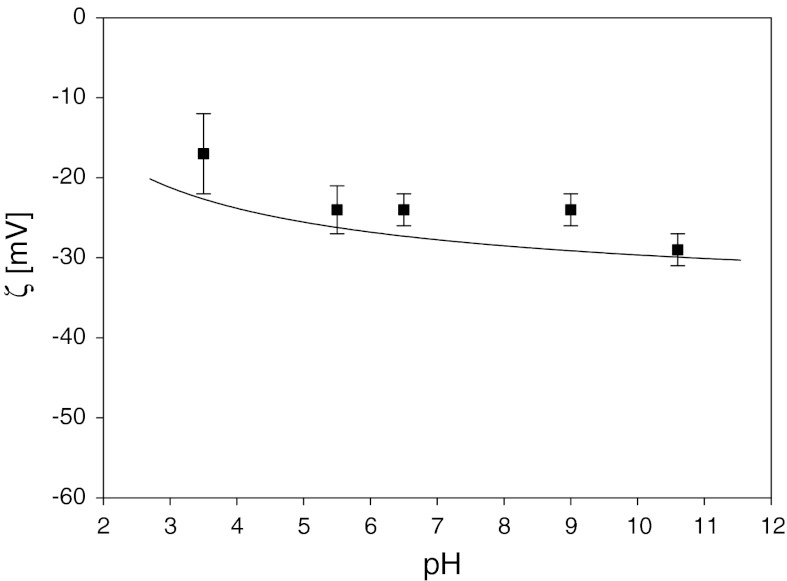
The dependence of the zeta potential determined by the streaming potential and electrophoretic measurements on pH (*I* = 10^−2^ M). Square points (*filled square*) represent the streaming potential results obtained from pH-cycle measurements. The *solid line* denotes the reference data obtained in the bulk (smoothened), i.e., $\zeta_{\infty } = \zeta_{\text{p}} /\sqrt 2$. The measurements were performed for the dense silver particle monolayer deposited at pH = 5.8 and *I* = 10^−2^ M

In Fig. 12 the dependence of the silver particle monolayer zeta potential on pH is shown.

The experimental procedure was as follows: first the silver monolayer having the coverage of 0.28 was obtained. Afterward, pH was changed in a discrete manner from pH = 3.5 to pH = 10.5. The streaming potential pertinent to a fixed pH value was simultaneously measured. The monolayer zeta potential was calculated from Eq. (1). A few pH cycles were performed, which showed that the zeta potential at each pH was constant within experimental error bounds. These results were compared with the reference values calculated from Eq. (7) using the bulk zeta potential of the silver particle. This is also depicted in Fig. 12 as a solid line. As can be noticed, the agreement of the ‘bulk’ and ‘surface’ values of the silver particle zeta potential is quite satisfactory for the entire range of pH (within standard deviation).

## Conclusions

Using a stable suspension of silver nanoparticles, homogeneous monolayers were produced in the self-assembly process, carried out under diffusion transport. The monolayer coverage, determined via AFM and SEM imaging, can be precisely regulated by the suspension concentration, adsorption time, and the ionic strength.

It was demonstrated that the coverage of the monolayers can be more efficiently determined via the in situ streaming potential measurements. The variations of the calculated zeta potential of the monolayers with the particle coverage were adequately interpreted in terms of the electrokinetic model expressed by Eqs. (5) and (6). This is of significance for basic science because the validity of the electrokinetic model to interpret the behavior of the conductive nanoparticle systems was confirmed for the first time.

Using this model, one can directly determine, via the experimental streaming potential data, the kinetics of particle adsorption and desorption under various transport conditions such as diffusion and forced convection (flow). In addition, using the electrokinetic studies, the characteristics of the monolayers can be acquired, in particular, one can determine their stability against pH cycling and ionic strength variations. This has a practical significance for optimizing conditions of stable silver nanoparticle film preparation on solid substrates.

## Highlights

- Silver particle monolayers of controlled density and structure were produced.
- The properties of monolayers were thoroughly characterized via in situ streaming potential measurements.
- A convenient method of determining coverage of silver nanoparticle films was developed.
